# Punicalagin Exerts Protective Effects against Ankylosing Spondylitis by Regulating NF-*κ*B-TH17/JAK2/STAT3 Signaling and Oxidative Stress

**DOI:** 10.1155/2020/4918239

**Published:** 2020-09-23

**Authors:** Xinzhe Feng, Qinyuan Yang, Chen Wang, Wenwen Tong, Weidong Xu

**Affiliations:** ^1^Department of Orthopedics, Changhai Hospital, Second Military Medical University, Shanghai, China; ^2^Department of Geriatrics, Huadong Sanatorium, Wuxi, China

## Abstract

**Background:**

Ankylosing spondylitis (AS) is a chronic inflammatory disease characterized by sacroiliitis and spinal rigidity of the axial joints. The role of oxidative stress and increased proinflammatory cytokines is well documented in AS pathogenesis. Punicalagin (2,3-hexahydroxydiphenoyl-gallagyl-D-glucose), an ellagitannin widely present in pomegranates, is found to exhibit potent anti-inflammatory, antiproliferative, and antioxidative effects. The present study was undertaken to investigate the effects of punicalagin in a rodent model of AS.

**Methods:**

BALB/c mice induced spondylitis were sacrificed 24 h after the last injection of proteoglycan extract. Histological scoring was done to assess the degree of the disease. The expression of JAK2/STAT3 proteins and proteins of the nuclear factor-*κ*B (NF-*κ*B) pathway was determined by immunoblotting. Serum levels of inflammatory mediators—TNF-*α*, IL-1*β*, IL-6, IL-17A, and IL-23—were assessed. Levels of lipid peroxidation and reactive oxygen species (ROS) were quantified. Antioxidant status as a measure of activities of antioxidant enzymes—catalase (CAT), glutathione peroxidase (GPx), and superoxide dismutase (SOD)—was determined.

**Results:**

Punicalagin effectively improved antioxidant status and decreased lipid peroxidation, ROS production, and serum levels of inflammatory mediators. NF-*κ*B pathway and JAK2/STAT3 signaling were significantly (*p* < 0.05) downregulated. Punicalagin effectively regulated the production of cytokines by the Th17 cells and the IL-17A/IL-23 axis.

**Conclusion:**

The observations suggest that punicalagin exerts a protective role in AS via reducing oxidative stress and regulating NF-*κ*B/TH17/JAK2/STAT3 signal. Punicalagin thus could be explored further as a potent candidate compound in the treatment of AS.

## 1. Introduction

Ankylosing spondylitis (AS) is a chronic, progressive inflammatory condition with a global prevalence rate of 0.1-1.4% [1]. AS primarily affects the lumbar spine and sacroiliac peripheral joints, eventually leading to bony ankylosis, pain, and disability [2]. Spinal fusion due to abnormal syndesmophyte formation limits spinal mobility and reduces the life quality of patients with AS [3, 4]. Studies have reported disturbed cytokine networks and elevated reactive oxygen species (ROS) levels in AS [5–7]. Proinflammatory cytokines have been involved in the stimulation of osteoclasts and the induction of TNF-*α* and various other inflammatory cells. However, the exact mechanism in the AS pathogenesis is still under investigation and seems to involve a variety of factors. Many studies have shown that cytokine network abnormality is an important feature of AS pathology [6, 7]. Of all the interleukins secreted by Th17 cells, IL-1*β*, IL-17, and IL-26 are most specific to Th17 cell response [2, 8–11]. NF-*κ*B signaling, one of the significant pathways of the inflammatory process, is found to be involved in the etiology of AS [12]. NF-*κ*B induces the expression of TNF-*α*, IL-1*β*, and other proinflammatory cytokines [13, 14]. TNF-*α* is found to induce further NF-*κ*B by positive feedback regulation by binding to TNF receptors (TNFR1 or TNFR2) [15]. Janus kinase (JAK) signaling is found to play a key role in bone development and metabolism [16]. Janus kinases 1/2/3 (JAK1/2/3) and tyrosine kinase 2 (TYK2), along with activators of transcription (STAT) signaling and signal transducers, are the major factors in the transmission of pro- and anti-inflammatory cytokine signals [17, 18]. The JAK2/TYK2-STAT3 signal is found to be involved in IL-23-mediated regulation of Th17 cells. Thus, JAK-2/STAT3 signaling could be considered a major target in the treatment of AS [19]. Increased levels of ROS have been reported to be involved in the pathogenesis of AS [20]. During the inflammatory process, cells as neutrophils produce raised levels of ROS that lead to oxidative stress. The elevated ROS further induce the production of inflammatory mediators as acute-phase proteins [21, 22].

Several plant-derived compounds have been shown to possess neuroprotective effects [5, 23, 24]. Punicalagin (2,3-hexahydroxydiphenoyl-gallagyl-D-glucose) is an ellagitannin and is the major bioactive polyphenol widely present in pomegranates. Punicalagins are water-soluble and hydrolyzed to smaller phenolic compounds. Punicalagin is found to exhibit potent anti-inflammatory, antioxidant, and antiproliferative effects [25, 26]. Previously, it was reported that punicalagin would be beneficial in some diseases, including Alzheimer's disease [27], memory impairment [28], and endometritis [29], which is due to its inhibitory effects on MAPK and NF-*κ*B activation. Punicalagin treatment effectively inhibits proinflammatory cytokine, nuclear factor *κ*B (NF-*κ*B), and mitogen-activated protein kinase (MAPK) expression *in vitro* in osteoporosis [30]. Punicalagin which has potent anti-inflammatory effects could be beneficial for ankylosing spondylitis like chronic progressive inflammatory conditions. However, no published study has fully investigated the effects of punicalagin on the development of AS. Therefore, the present work studies the effects of systemic administration of punicalagin in AS-induced mice to determine whether punicalagin administration can relieve ankylosing spondylitis in an AS mouse model.

## 2. Materials and Methods

Antibodies against JAK2, STAT3, p-JAK2, and p-STAT3 were procured from Cell Signaling Technology (Danvers, MA, USA). TNF-*α*, NF-*κ*B p65, I*κ*B*α*, p-I*κ*B*α*, p-IKK*α*, IKK*α*, p-IKK*β*, IKK*β*, horseradish peroxidase-labeled IgG secondary antibodies, and *β*-actin were purchased from Santa Cruz Biotechnology (Texas, USA). Punicalagin and the buffers used in expression studies were purchased from Sigma-Aldrich (St. Louis, MO, USA). ELISA kits for the determination of cytokines—TNF-*α*, IL-1*β*, IL-6, IL-17A, and IL-23—were obtained from BioLegend (San Diego, CA, USA). Levels of ROS production and lipid peroxidation (malondialdehyde content) were measured using kits from Abcam, USA. Superoxide dismutase (SOD), catalase (CAT), and glutathione peroxidase (GPx) enzyme activities were assessed using kits from Abcam, USA. All other reagents and chemicals used in the study were procured from Sigma-Aldrich, otherwise mentioned.

### 2.1. Experimental Animals

BALB/c mice (male, 25-30 g; *n* = 60) were sourced from the University Laboratory Animal Facility. The experimental design and study protocols were reviewed and approved by the Ethical Committee of the University. The animals were handled with the utmost care, and the experiments were conducted following the National Guidelines of the Animal Care Committee in acquiescence with the International Guidelines for the Study of Laboratory Animals. The animals were kept in sterile cages (*n* = 6 per age) under controlled animal house conditions (12 h light/dark cycle, 23 ± 2°C, 55-60% relative humidity). The mice were provided unlimited access to commercially available standard mice pelleted diet and water. The mice were accustomed to the animal house environment for five days before the initiation of the study.

### 2.2. Study Design

Sixty mice were separated to 6 treatment groups as follows: (i) normal control—mice received standard pellet diet; (ii) ankylosing spondylitis group (AS control)—mice induced with AS and received a standard pellet diet; and (iii-v) punicalagin treatment groups——mice induced with AS and received the standard pellet diet and 12.5 mg/kg, 25 mg/kg, and 50 mg/kg of punicalagin, respectively. The doses were chosen based on the results obtained from previous studies conducted in our laboratory. Punicalagin was administered orally every day for 15 days starting day 1 after the induction of AS. (vi) In the punicalagin control group, normal mice received a standard diet and 50 mg/kg punicalagin.

### 2.3. Induction of AS

AS was induced in mice by injecting (intraperitoneally) human proteoglycan extract (2 mg) dissolved in dimethyldioctadecylammonium (DDA; 2 mg). The proteoglycan extract was administered 4 times in two weeks at an interval of 4 days [31]. Following 2 weeks after the 1^st^ injection, the animals exhibited symptoms of axial skeleton ankyloses. The animals were then scored for signs and symptoms of arthritis as follows: 0—no symptoms, 1—swelling and reddishness in one of the toes, 2—exhibiting swelling and redness in more than one toe, 3—stiffness observed in toes, and 4—deformity in the toes or ankle involvement. The total score for each mouse was calculated from the average of scores obtained for each toe [32]. After 24 h of final injection and scoring for symptoms, the animals were sacrificed, the blood was collected, and the serum was separated and used for analysis.

### 2.4. Histological Analysis

The tissue removed from the vertebra was exposed to hematoxylin and eosin (H&E) staining. The extent of the inflammation and spinal damage was scored as 0 to 4 as described previously ([33]): score 1—presence of inflammatory cells permeating the annulus fibrosus and around the intervertebral disc (IVD), 2—less than 50% of the IVD which are affected, 3—greater than 50% of the IVD which are affected, and 4—presence of bony fusion/fusion of the cartilage in at least 18-22 IVDs (from the cervical region of the lumbar area).

### 2.5. Detection of Oxidative Stress and Antioxidant Enzymes

Tissues (connective) excised from the vertebra (*n* = 6/group) were homogenized in ice-cold PBS and centrifuged at 4°C at 1000 g force for 15 min. Activities of antioxidant enzymes (SOD, CAT, and GPx), lipid peroxidation levels (MDA content), and ROS production levels were determined in the supernatant collected. The total protein content in the supernatant obtained was quantified by the bicinchoninic acid (BCA) method (protein assay kit, Sigma-Aldrich, USA). The DCF ROS/RNS Assay Kit (Abcam) was used to quantify ROS levels. Dichlorodihydrofluorescein DiOxyQ (DCFH-DiOxyQ), a fluorogenic probe specific to ROS/RNS, was used in the assay. The fluorescence intensity reflects the levels of ROS/RNS present in the sample. The intensity was measured at 480 nm excitation/530 nm emission using Synergy™ 2 Multi-function Microplate Reader.

The activity of enzymes SOD, CAT, and GPx was determined by ELISA following instructions specified by the manufacturer. Lipid peroxidation levels in terms of malondialdehyde (MDA) content were measured using a lipid peroxidation assay kit (Abcam, USA), and the levels were expressed as nM MDA formed/mg of protein.

### 2.6. Determination of Levels of Cytokine and NO

The inflammatory cytokine levels of TNF-*α*, IL-6, Il-17A, IL-1*β*, and IL-23 in the serum were determined by ELISA using kits from BioLegend according to the manufacturer's protocol. Levels of NO were evaluated as nitrite accumulation using Griess reagent (NO assay kit, Abcam).

### 2.7. Immunoblotting

Protein expression analysis was performed at 24 h after the final injection of proteoglycan extract. The excised vertebral tissues were homogenized using cell lysis buffer from Cell Signaling Technology, USA [20 mM Tris-HCl (pH 7.5), 1 mM EGTA, 1 mM Na_2_EDTA, 1% Triton, 150 mM NaCl, 2.5 mM sodium pyrophosphate, 1 mM Na_3_VO_4_, 1 *μ*g/mL leupeptin, and 1 mM beta-glycerophosphate]. The whole-cell extracts obtained were subjected to centrifugation (1000 g-force, 15 min) at 4°C. The collected supernatant was used for analysis. Cytosol and nuclear fractions were separated from the whole-cell homogenate using ReadyPrep™ Protein Extraction Kit (cytoplasmic/nuclear) from Bio-Rad (CA, USA). The total protein contents of the whole-cell extracts and in the cytosol and nuclear fractions were quantified using kits from Thermo Fisher Scientific. Protein samples of equal concentration (60 *μ*g) from whole cell, cytoplasmic, and nuclear extracts were loaded on 12% SDS-PAGE gels and electrophoretically separated. The size-fractionated protein bands obtained were blot-transferred onto polyvinylidene difluoride (PVDF) membranes (Thermo Fisher Scientific). The blotted membranes were blocked using 5% skimmed milk for 60 min to exclude any endogenous peroxidase activity. Membranes washed with Tris-buffered saline and Tween-20 (TBST) were treated with primary antibodies at 4°C overnight. Following incubation, the membranes were washed with TBST and incubated further with secondary antibody (horse radish peroxidase-conjugated; 1 : 2000, Santa Cruz Biotechnology) at room temperature for 60 min. Post washing with TBST, the bands were visualized using an enhanced chemiluminescence system (Millipore, USA). The positive bands obtained were scanned and analyzed using ImageJ software (SuperSignal; Pierce, IL, USA).

### 2.8. Statistical Analysis

The results of the present study were statistically analyzed (SPSS software, Version 21.0, IBM Corporation, USA). The data were analyzed by one-way analysis of variance (ANOVA) and Duncan's multiple range test (DMRT). Values at *p* < 0.05 were regarded as statistically significant.

## 3. Results

### 3.1. Punicalagin Reduces the Severity of Disease Progression

**Figure 1:**
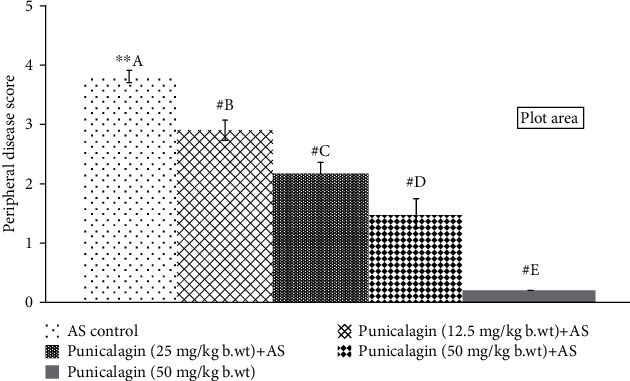
Punicalagin reduces peripheral disease progression. The results are presented as the mean ± SD (*n* = 6). # represents *p* < 0.05 vs. AS control. A–E represent the mean from different treatment groups that differ at *p* < 0.05.

Following 24 h of the last injection, the mice were observed for symptoms of arthritis and ankyloses. The mice exhibiting signs and symptoms of arthritis and axial skeleton ankyloses were scored from 1 to 4 accordingly. The AS control group animals presented with scores of 3.8 ± 0.10 (Figure 1). Punicalagin administration resulted in significantly lower scores (*p* < 0.05). 50 mg/kg punicalagin-administered mice presented scores of 1.5 ± 0.08. Punicalagin treatment prevented stiffness of deformity in the toes.

### 3.2. Effects of Punicalagin on the Extent of the Inflammation and Spinal Damage

**Figure 2:**
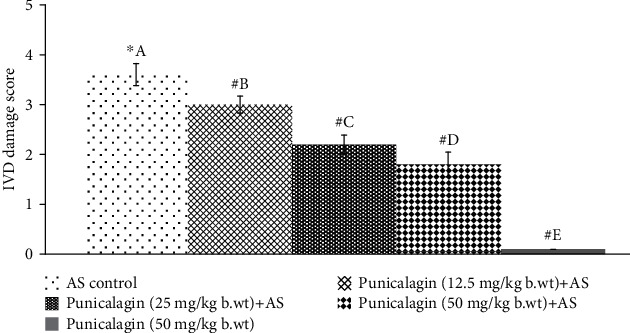
Punicalagin reduced IVD damage progression. The results are presented as the mean ± SD (*n* = 6). # represents *p* < 0.05 vs. AS control. A–E represent the mean values of experimental groups that differ at *p* < 0.05.

Histological analysis revealed the extent of spinal damage. Systemic supplementation of punicalagin to AS-induced mice was found to significantly (*p* < 0.05) delay the progression of the peripheral disease as shown in Figure 2. The scores of H&E-stained intervertebral joints showed significant infiltration of inflammatory cells into the IVD area following AS induction. The observations also revealed the fusion of the cartilage. Punicalagin at all three tested doses substantially reduced the infiltration of inflammatory cells and prevented damage. AS control animals presented with scores of 3.6 ± 0.22 vs. 1.8 ± 0.07 in mice treated with 50 mg punicalagin.

### 3.3. Punicalagin Reduces Oxidative Stress

**Figure 3:**
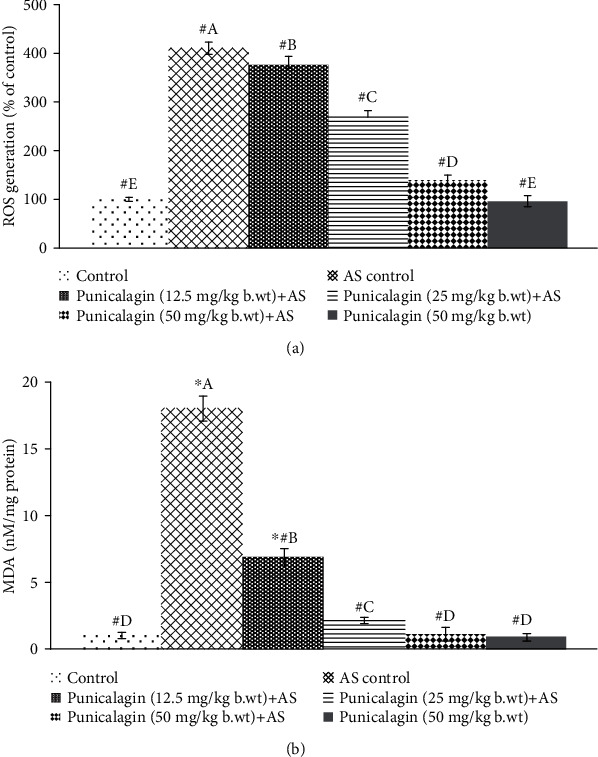
Punicalagin reduced ROS generation following AS induction (a) and MDA levels (b). The results are presented as the mean ± SD (*n* = 6). ∗ represents *p* < 0.05 vs. normal control; # represents *p* < 0.05 vs. AS control. A–E represent the mean values from the experimental groups that differ at *p* < 0.05.

Oxidative stress is well established in the pathogenesis of AS [5]. ROS and lipid peroxidation levels were measured in vertebral tissues 24 h after the last injection of AS. A significant increase in ROS and MDA levels was observed in AS-induced mice. As shown in Figures 3(a) and 3(b). ROS generation increased multifold (410.6%) in the AS control vs. the normal control group. MDA content was observed to rise to 18.20 nM/mg protein in AS control mice vs. 1.06 nM/mg protein in the normal control. Interestingly, treatment with punicalagin significantly decreased (*p* < 0.05) ROS levels. The 50 mg and 25 mg doses were found to be more effective in decreasing ROS levels in comparison to a lower dose of 12.5 mg. Consistent with the ROS levels, MDA content was also significantly (*p* < 0.05) reduced in punicalagin treatment in comparison to AS control. The punicalagin alone-treated group was presented with ROS and MDA levels close to normal control.

### 3.4. Punicalagin on the Activities of Antioxidant Enzymes

**Figure 4:**
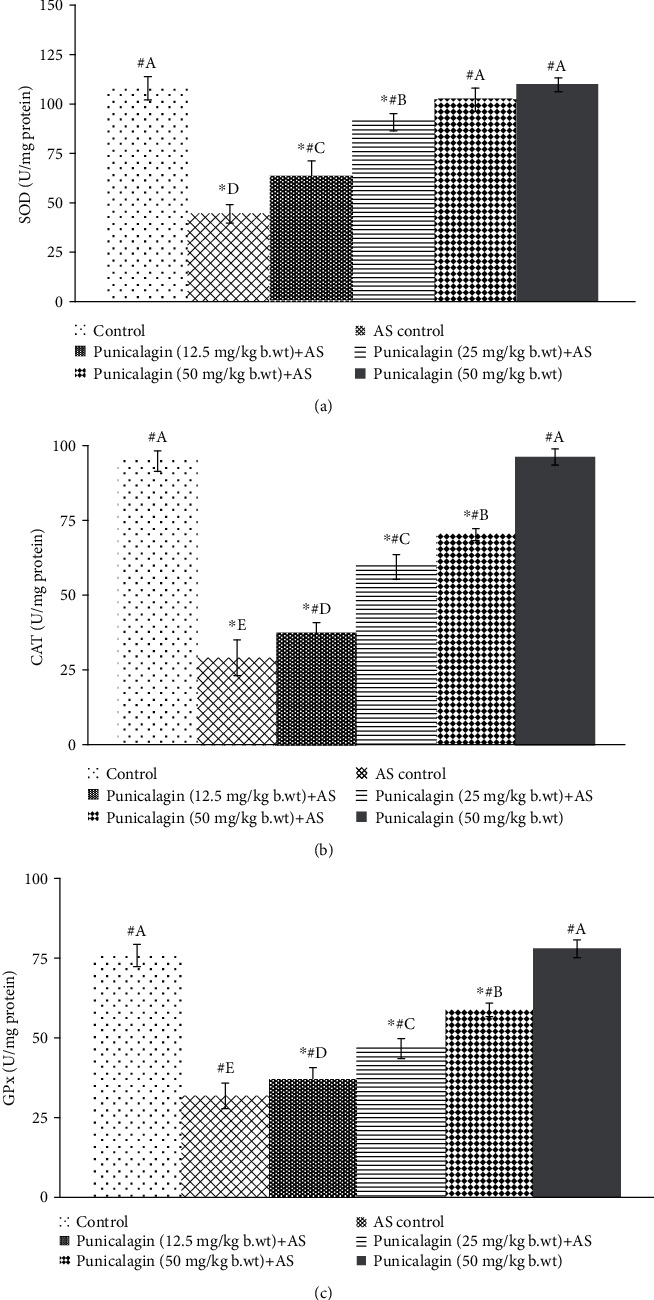
Punicalagin enhances the activities of antioxidant enzymes: SOD (a), CAT (b), and GPx (c). The results are presented as the mean ± SD (*n* = 6). ∗ represents *p* < 0.05 vs. normal control; # represents *p* < 0.05 vs. AS control. A–E represent the mean values from different experimental groups that differ at *p* < 0.05.

The activities of SOD, CAT, and GPx were assessed following AS induction. Figures 4(a)–4(c) illustrate the significantly (*p* < 0.05) declined activities of enzymes in AS control animals vs. the enzyme activities observed in normal control mice. In AS control, the activities of SOD, CAT, and GPx were decreased significantly. Punicalagin markedly (*p* < 0.05) enhanced the enzyme activities of SOD, CAT, and GPx activities which were increased.

### 3.5. Punicalagin Regulated NF-*κ*B Signaling Cascade

**Figure 5:**
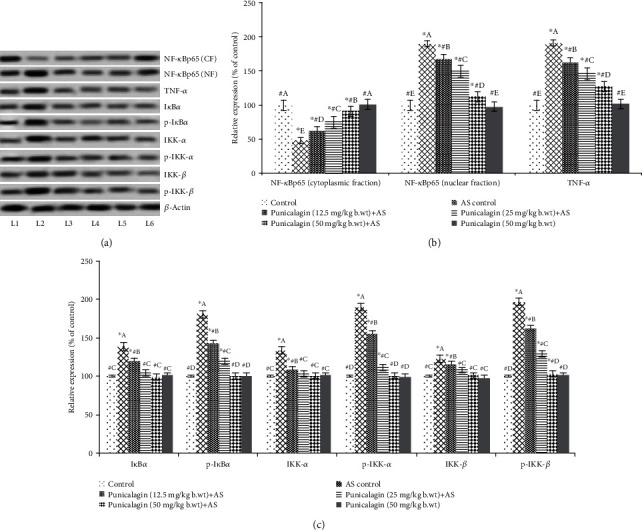
Punicalagin regulates NF-*κ*B signaling. Punicalagin downregulated activation of NF-*κ*B (a, b) and inhibitor kinases of NF-*κ*B signaling following AS (a, c). Representative immunoblot. (a) Expression levels of test proteins relative of control expressions set at 100% (the expression of phosphorylated proteins was normalized by respective nonphosphorylated protein expression). The results are presented as the mean ± SD (*n* = 6). ∗ represents *p* < 0.05 vs. normal control; # represents *p* < 0.05 vs. AS control. A–E represent the mean values from different experimental groups that differ at *p* < 0.05. L1: control; L2: AS control; L3: punicalagin (12.5 mg/kg body weight)+AS; L4: punicalagin (25 mg/kg body weight)+AS; L5: punicalagin (50 mg/kg body weight)+AS; L6: punicalagin (50 mg/kg body weight).

Oxidative stress conditions are known to activate NF-*κ*B signaling, and proinflammatory cytokines present in AS are also important/classical activators of the NF-*κ*B signaling pathway. The pathway is found to be associated with AS pathogenesis. AS induction caused activation of (*p* < 0.05) nuclear NF-*κ*B (p65) with substantially (*p* < 0.05) lower cytosolic levels of NF-*κ*B (p65) (Figures 5(a) and 5(b)). The observations reveal activation of NF-*κ*B as reflected by upregulated levels of TNF-*α* and levels of phosphorylation of the regulatory kinases such as IKK*α*, IKK*β*, and I*κ*B*α* (Figures 5(a)–5(c)). Punicalagin supplementation resulted in a significant suppression (*p* < 0.05) of NF-*κ*B p65 (nuclear fraction) expression vs. AS control. 50 mg dose of punicalagin significantly (*p* < 0.05) inhibited NF-*κ*B p65 levels in the nuclear fraction. The expressions were found to be decreased from 190.2% to 112.3%, while 25 mg punicalagin reduced to 149.7%. Punicalagin at the tested doses of 12.5 mg/kg, 25 mg/kg, and 50 mg/kg significantly (*p* < 0.05) downregulated phosphorylation of regulatory kinases IKK*α*, IKK*β*, and I*κ*B*α* vs. the AS control group. Enhanced TNF-*α* observed in AS reduced to 127.34% from 191.35% (*p* < 0.05, 50 mg punicalagin vs. AS control) in animals administered with punicalagin. In expression analysis of phosphorylated proteins, the expression of p-protein was normalizing by the respective non-p-protein expression, and so the increase or decrease phosphorylation is due to pathway modulation. These observations suggest the effective inhibition of NF-*κ*B signaling by punicalagin.

### 3.6. Punicalagin Reduced Serum Cytokine Levels

**Figure 6:**
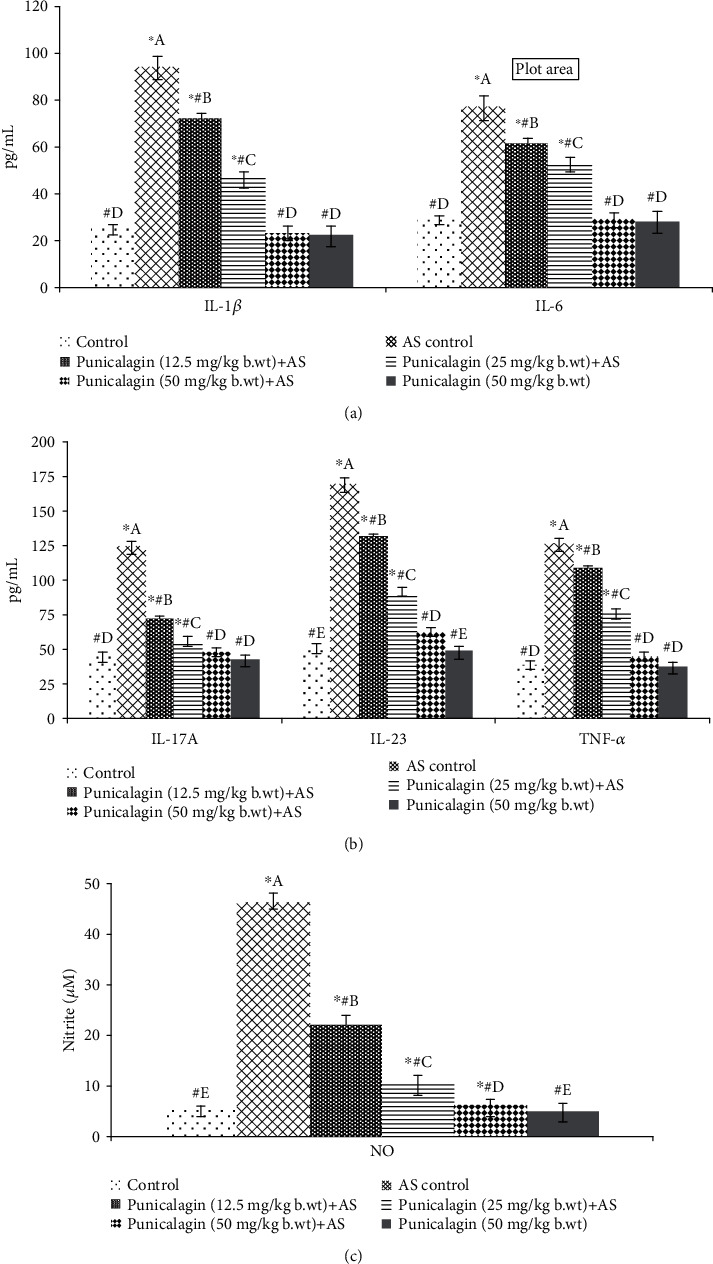
Effects of punicalagin on serum levels of inflammatory mediators. Punicalagin reduced the levels of inflammatory cytokines (a), the activity of Th17 cells (b), and the levels of NO (c). The results are represented as the mean ± SD (*n* = 6). ∗ represents *p* < 0.05 vs. normal control; # represents *p* < 0.05 vs. AS control. A–E represent the mean values from different experimental groups that differ at *p* < 0.05.

Significantly (*p* < 0.05) elevated serum levels of cytokines—IL-1*β*, IL-6, 1L-17A, IL-23, and TNF-*α*, were noticed in AS induction (Figure 6). Punicalagin-mediated inhibition of NF-*κ*B signaling cascade was reflected by markedly (*p* < 0.05) declined serum levels of IL-1*β*, IL-6, and TNF-*α*. The serum levels of IL-1*β*, IL-6, and TNF-*α* were observed to be decreased significantly (*p* < 0.05) on treatment with punicalagin. The levels of IL-17A and IL-23 were markedly (*p* < 0.05) reduced on punicalagin supplementation. Serum levels of NO were substantially reduced by punicalagin in a dose-dependent fashion. The observations suggest the effective downregulation of the inflammatory mediators by punicalagin. 50 mg dose was noted to exert maximal anti-inflammatory effects in comparison to the other two doses studied.

### 3.7. Punicalagin Regulates the JAK2/STAT3 Signal

**Figure 7:**
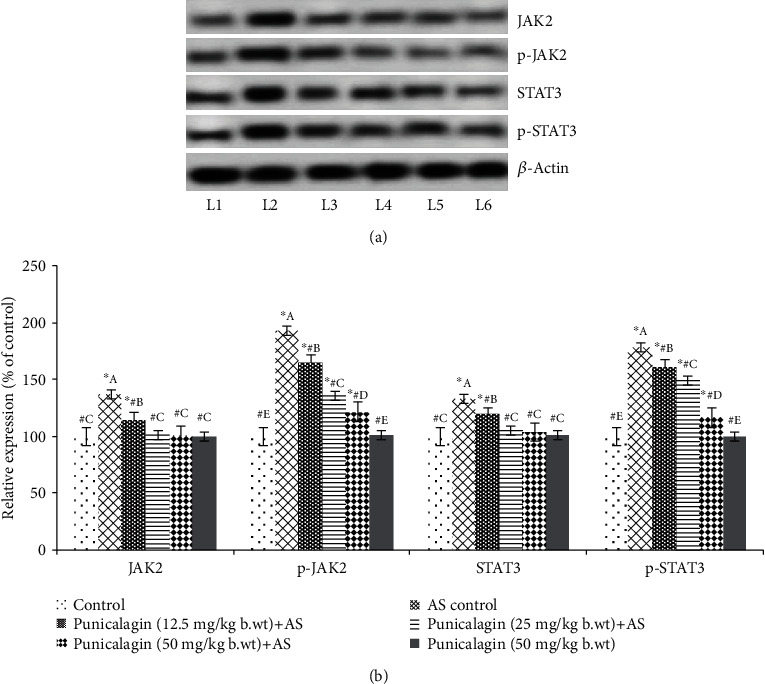
Punicalagin regulated the JAK2/STAT3 signal. Representative immunoblot. (a) Expressions of test proteins relative to control expressions set at 100% (the expression of phosphorylated proteins was normalized by respective nonphosphorylated protein expression). The results are represented as the mean ± SD (*n* = 6). ∗ represents *p* < 0.05 vs. control; # represents *p* < 0.05 vs. AS control. A–E represent the mean values from different experimental groups that differ at *p* < 0.05. L1: control; L2: AS control; L3: punicalagin (12.5 mg/kg body weight)+AS; L4: punicalagin (25 mg/kg body weight)+AS; L5: punicalagin (50 mg/kg body weight)+AS; L6: punicalagin (50 mg/kg body weight).

The influence of punicalagin on the JAK2/STAT3 signaling was assessed by analyzing the protein expressions by western blotting. JAK2/STAT3 signaling was found to be upregulated following induction of AS as reflected by significantly (*p* < 0.03) elevated phosphorylation of JAK2 and STAT3 (Figure 7). The expressions of total JAK2 and STAT3 though were also substantially upregulated vs. normal control. Interestingly, the activation of the pathway was significantly (*p* < 0.05) downregulated by systemic administration of punicalagin for 15 days. Increased phosphorylation levels of JAK2 and pSTAT3 AS control mice were decreased on treatment with punicalagin. Mice administrated with punicalagin alone presented almost near control levels of p-JAK and p-STAT3 expressions. The results suggest that punicalagin effectively regulated the JAK2/STAT3 signal.

## 4. Discussion

Inflammatory responses implicated in the pathogenesis of ankylosing spondylitis (AS) lead to erosive osteopenia and unusual bony overgrowth [34]. Infiltration of inflammatory cells such as macrophages and lymphocytes, joint fibrosis, synovial thickening, and bone ankyloses is observed in the joints [23, 35]. Considering the delay in diagnosis and insufficient effective therapeutic options available, identification and development of more effective compounds become inevitable. Recently, much research has been focused on the efficacy of plant-derived compounds in AS [24].

In this study, we investigated the effects of systemic administration of punicalagin in AS-induced mice. Punicalagin, an ellagitannin that is the most abundant polyphenol, shows significant pharmacological activities, including antioxidant, anticancer, and anti-inflammatory activities. The results of the study demonstrated that punicalagin administration significantly decreased the progression of the disease and decreased the damage of the intravertebral discs (IVD). Further, a remarkable reduction in the levels of ROS and lipid peroxidation (MDA) was noticed in punicalagin treatment. Oxidative stress has been well documented as a major factor in the pathogenesis of AS and reported to be involved in elevated MDA levels in patients with AS compared to healthy individuals [5, 20, 21]. SOD, CAT, and GPx are the important antioxidant enzymes that play crucial roles in scavenging superoxide radicals in cells, thus reducing radical-induced damage to the biomolecules and other cellular components [21]. Decreased activities of the enzymes observed in AS could be due to the excessive ROS. The excessive and uncontrolled elevated levels of ROS lead to oxidative stress conditions [36]. Punicalagin effectively improved the activities of all these enzymes such as CAT, GPx, and SOD which could have in part contributed to the reduced levels of ROS and MDA content. The significant decrease in ROS production levels could also be due to the direct antioxidant activity of punicalagin.

Abnormal or aberrant inflammatory cytokine pathways have been well documented in the pathogenesis of AS [37]. Here, we assessed the effects of punicalagin on NF-*κ*B signaling and TNF-*α*, IL-1*β*, IL-6, IL-23, and IL-17. NF-*κ*B, a chief transcription factor, is well documented in the regulated expression of many proteins that are involved in immune responses [38]. Here, we noticed significantly upregulated NF-*κ*B activation as reflected by the increased expression of NF-*κ*B p65 in nuclear fraction along with elevated levels of IL-1*β*, IL-6, and TNF-*α* in the serum of AS-induced mice. A previous study by Liu et al. [24] has also demonstrated increased NF-*κ*B p65 and raised levels of IL-1*β*, IL-6, and TNF-*α* in AS. Punicalagin mediated significantly downregulated phosphorylation of regulatory proteins (I*κ*B*α*, IKK*α*, and IKK-*β*) which consequently could have resulted in suppressed activation of NF-*κ*B. Suppression activation of NF-*κ*B signaling is beneficial as this leads to the decline in the expressions of several inflammatory response genes regulated by NF-*κ*B [24]. NO is known to play a significant role in inflammatory and immune responses [39]. Punicalagin-mediated reduced TNF-*α*, IL-1*β*, IL-6, and NO serum levels in AS-induced mice illustrate the anti-inflammatory efficacy. Further, it has been shown that Janus kinases (JAK) are major transducers of cytokine signals in Th17 cells [40, 41]. Th17 cells, which is a key player in AS, secrete various inflammatory mediators such as IL-17, IL- 6, IL-22, IL-26, interferon- (IFN-) *γ*, and TNF-*α* [8]. Aberrantly, activation of IL-23/IL-17 cytokine axis has been noticed in AS [42]. Melis et al. [43] reported raised serum IL-23 levels in AS. It has been shown that IL-23R exerts its effects via the JAK2/STAT3 signaling upon binding of IL-23 [19]. IL-23 is regarded as a dominant factor in the inactivation of the JAK2/STAT3 pathway. The JAK-STAT pathway plays a vital role in signal transduction activated by growth factors and cytokines [44]. Our findings illustrated that punicalagin treatment interfered with the NF-*κ*B and MAPK pathways, including JNK and STAT phosphorylation. In immunity and inflammation, the NF-*κ*B and MAPK pathways play an important role in regulating the gene levels of the primary proinflammatory mediators [45–47]. Interestingly, punicalagin-mediated downregulated phosphorylation of JAK2 and STAT3 indicates inhibition of the JAK2/STAT3 signal. A marked decrease in IL-23 and IL-17A levels in the serum of AS-induced mice was observed on punicalagin treatment which in part could have contributed to the reduced levels of serum IL-17A. Also, Mathur et al. [48] reported that STAT3 phosphorylation is considered an important event in IL-6 signaling. Thus, punicalagin-mediated downregulation of the JAK2/STAT3 signal and IL-23 levels could have contributed to the decrease in IL-17 levels. The results illustrate that punicalagin effectively reduced the levels of ILs and inflammatory responses. These observations propose the potent anti-inflammatory roles of punicalagin.

### 4.1. Limitation of the Study

We do mention here that the DCF ROS/RNS assay kit which was used to quantify ROS/RNS which is meant to use for cell lysates, biofluids, and culture supernatant. In our study, the homogenization and centrifugation processes were necessary to prepare the tissue (connective) samples excised from the vertebra. Considering the very unstable nature of ROS/RNS and prolonged homogenization, there is a possibility that we would not have achieved the very accurate measurement of ROS. However, results stand valid and significant as all the samples were processed in similar manners without any variation.

## 5. Conclusion

Punicalagin was found to exert antioxidative and anti-inflammatory effects via reducing ROS and lipid peroxidation levels and by regulating the major pathways of inflammatory response of NF-*κ*B and JAK2/STAT3 signaling pathways. Punicalagin could be proved to be a therapeutic option for ankylosing spondylitis and other chronic inflammatory diseases.

## Data Availability

All data are provided in this study, and raw data can be requested to the corresponding author.
